# Four-Dimensional Magnetic Resonance Pulmonary Flow Imaging for Assessing Pulmonary Vasculopathy in Patients with Postcapillary Pulmonary Hypertension

**DOI:** 10.3390/jcm14030929

**Published:** 2025-01-31

**Authors:** Jorge Nuche, Inés Ponz, Violeta Sánchez Sánchez, Javier Bóbeda, Ángel Gaitán, Karen López-Linares, María Dolores García-Cosío, Fernando Sarnago Cebada, Javier Sánchez González, Fernando Arribas Ynsaurriaga, Jesús Ruíz-Cabello, Borja Ibáñez, Juan F. Delgado

**Affiliations:** 1 Cardiology Department, Hospital Universitario 12 de Octubre, Instituto de Investigación Sanitaria Hospital 12 de Octubre (imas12), Servicio de Cardiología, Avenida de Córdoba s/n, 28041 Madrid, Spain; jorge-nuche@hotmail.com (J.N.); violetasan@gmail.com (V.S.S.); lolagcosio@gmail.com (M.D.G.-C.); fernando.sarnago@salud.madrid.org (F.S.C.); fernando.arribas@salud.madrid.org (F.A.Y.); 2 Centro de Investigación Biomédica En Red de Enfermedades CardioVasculares (CIBERCV), 28029 Madrid, Spain; bibanez@cnic.es; 3 Centro Nacional de Investigaciones Cardiovasculares, Calle de Melchor Fernández Almagro 3, 28029 Madrid, Spain; 4 Cardiology Department, Hospital Universitario La Paz, Instituto de Investigación IDIPAZ, Paseo de la Castellana 261, 28029 Madrid, Spain; 5 Vicomtech, Basque Research and Technology Alliance (BRTA), Paseo Mikeletegi 57, 20009 San Sebastián, Spain; jbobeda@vicomtech.org (J.B.); klopez@vicomtech.org (K.L.-L.); 6 Medical Physics Department, Hospital Universitario 12 de Octubre, Instituto de Investigación Sanitaria Hospital 12 de Octubre, Avenida de Córdoba s/n, 28041 Madrid, Spain; angel.gaitan@salud.madrid.org; 7 eHealth Group, Biogipuzkoa Health Research Institute, Paseo Dr. Beguiristán s/n, 20014 San Sebastián, Spain; 8 Philips Healthcare Ibérica, Calle María de Portugal, 1, 28050 Madrid, Spain; 9 Universidad Complutense de Madrid, Plaza de Ramón y Cajal s/n, 28040 Madrid, Spain; jruizcabello@cicbiomagune.es; 10 CIC BiomaGUNE, Basque Research and Technology Alliance (BRTA), Spain & Ikerbasque, Basque Foundation for Science, Kurutz Gain Industrialdea, 48009 Bilbao, Spain; 11 Centro de Investigaciones Biomédicas en Red de Enfermedades Respiratorias (CIBERES), Calle de Melchor Fernández Almagro 3, 28029 Madrid, Spain; 12 IIS-Fundación Jiménez Díaz, Avenida de los Reyes Católicos 2, 28040 Madrid, Spain

**Keywords:** heart failure, pulmonary hypertension, magnetic resonance, four-dimensional flow

## Abstract

**Background**: Noninvasive techniques for diagnosing combined postcapillary pulmonary hypertension (CpcPH) are unavailable. **Objective**: To assess the diagnostic performance of cardiac magnetic resonance (CMR)-based four-dimensional (4D)-flow analysis in identifying CpcPH. **Methods**: Prospective observational study of heart failure (HF) patients with suspected pulmonary hypertension (PH) who underwent simultaneous CMR and right heart catheterization. The 4D-flow biomarkers were calculated using an automatic pipeline. A predictive model including 4D-flow biomarkers associated with CpcPH with a *p*-value < 0.20 was built to determine the diagnostic performance of 4D-flow analysis to identify CpcPH. **Results**: A total of 46 HF patients (55.4 ± 14 years, 63% male) with confirmed PH (19 [41%] isolated postcapillary PH [IpcPH], 27 [59%] CpcPH) were included. No differences were found in baseline characteristics, echocardiography, or CMR anatomical and functional parameters, except for a higher Doppler-estimated systolic pulmonary pressure and larger pulmonary artery in CpcPH patients. The 4D-flow CMR analysis was performed in 31 patients (67%). The maximal peak velocity (67.1 [62.2–77.5] cm/s—IpcPH vs. 58.2 [45.8–66.0] cm/s—CpcPH; *p* = 0.021) and maximal helicity (339.9 [290.0–391.8]) cm/s^2^—IpcPH vs. 226.0 (173.5–343.7) cm/s^2^—CpcPH; *p* = 0.026) were significantly lower in patients with CpcPH. A maximal multivariable model including sex, maximal average, and peak velocities, Reynolds number, flow rate, and helicity showed fair diagnostic performance (area under the curve: 0.768 [95%-CI: 0.572–0.963]; sensitivity: 100%; specificity: 55%). **Conclusions**: In HF patients with PH, 4D-flow-derived maximal peak velocity and maximal helicity were significantly lower in CpcPH patients. A multiparametric model including maximal 4D-flow-derived biomarkers showed good diagnostic performance for identifying CpcPH.

## 1. Introduction

Heart failure (HF) is a highly prevalent disease associated with high morbidity and mortality and it significantly impacts healthcare expenditure [1]. The development of pulmonary hypertension (PH) is a common complication of chronic HF and is associated with worse outcomes and increased mortality [2].

PH in patients with HF, or group 2 pulmonary hypertension (PH2) [3] debuts as a passive process, secondary to the chronic increase in left ventricular preload that is retrogradely transferred to the pulmonary vasculature (isolated postcapillary PH [IpcPH]). This increased pulmonary vasculature pressure triggers a neurohormonal response leading to the development of pulmonary vascular remodeling consisting of a combination of endothelial dysfunction, a hyperproliferative status, and a chronic inflammatory response [4]. At this point, PH becomes an active phenomenon in which increased pulmonary artery (PA) pressure does not respond solely to the passive transmission of increased pressure in the left heart chambers but also to an intrinsic pulmonary vascular remodeling that actively contributes to the increased pulmonary pressures (combined postcapillary PH [CpcPH]) [3]. The development of CpcPH is associated with a poorer prognosis, independently of the HF etiology or type, and may contraindicate a heart transplant in patients with advanced HF [5].

Different noninvasive techniques are employed in the evaluation of PH patients (transthoracic echocardiogram, computed tomography, cardiac magnetic resonance [CMR]) and provide valuable information for the diagnosis and management of these patients. Although echocardiography provides information about PA pressure, it cannot distinguish between patients who have developed pulmonary vascular remodeling and those with isolated retrograde PH since no reliable method to estimate left ventricle (LV) filling pressures has been properly validated. This, the only available tool for the diagnosis of CpcPH is the invasive measurement of pulmonary pressures and vascular resistance through a right heart catheterization (RHC), with no noninvasive techniques allowing us to distinguish it from IpcPH [3].

The CMR-based four-dimensional (4D) flow sequence has emerged as a promising technique for evaluating flow patterns and vascular compliance in PH patients. This study aimed to determine the role of CMR-based 4D flow in identifying HF patients who have developed CpcPH.

## 2. Methods

This study’s rationale, design, and methodology have been thoroughly described previously [6]. The methodology for the primary objective of the study is outlined below.

### 2.1. Hypotheses and Objectives

We hypothesized that the pathobiological changes in the pulmonary vasculature of patients with CpcPH would lead to changes in PA blood flow that can be assessed by analyzing CMR-based 4D-flow sequences.

This study aimed to determine whether CMR-based 4D-flow analysis could be an appropriate tool to identify HF patients who have developed CpcPH.

### 2.2. Pulmonary Hypertension Definition

The definition of PH has been recently revised [3]. The updated criteria define PH following the invasive measurement of a mean PA pressure of >20 mmHg. The diagnosis of PH2 requires a PA wedge pressure of >15 mmHg. A pulmonary vascular resistance of ≥2 Wood units defines the presence of CpcPH.

When this study was initiated, the mean PA pressure threshold defining PH was 25 mmHg [7] and the pulmonary vascular resistance limit to distinguish between IpcPH and CpcPH was 3 Wood units. The former and current definitions for PH are presented in Supplementary Table S1.

For this analysis, all the results correspond to the former PH definitions, unless indicated otherwise.

### 2.3. Study Design and Population

This was a single-center, prospective, and observational study that included patients with PH2, according to the former definition of PH [7]. Patients with chronic HF with or without a recent acute decompensation; and reduced, mild-reduced, or preserved ejection fraction were eligible for the study [8]. Patients suspected of having PH based on echocardiographic assessments were screened for inclusion. Patients fulfilling all inclusion criteria and none of the exclusion criteria underwent RHC to confirm the diagnosis of PH2. Patients with confirmed PH2 were scheduled for a CMR with 4D flow acquisition within 24 h after the RHC (Supplementary Figure S1, Supplementary Table S2). All patients were treated according to current guideline recommendations.

All baseline information, echocardiographic parameters, and RHC- and CMR-derived measurements were recorded in a database.

The Institutional Ethics Committee of the University Hospital 12 de Octubre and the Spanish National Cardiovascular for Cardiovascular Research (CNIC) reviewed the study protocol and wrote informed consent forms. All patients signed informed consent before their inclusion in the study.

### 2.4. Right Heart Catheterization

RHC was performed under stable clinical conditions. Patients screened during an HF acute decompensation were clinically optimized before undergoing the RHC. For the RHC, a 7 French sheath was canalized through the right cephalic or basilic vein when possible. For those patients with unsuccessful peripheral vein canalization, the internal jugular vein followed by the common femoral vein were chosen as access. A Swan–Ganz catheter was then inserted into the PA and the pressure was recorded. The PA wedge pressure was measured at end-expiration and at the beginning of the QRS interval. All RHC measurements were performed by medical practitioners with PH expertise. The pressure curves obtained from the RHC were afterwards assessed by a single investigator (F.S.C.) who was responsible for the validation of the hemodynamic data.

### 2.5. Cardiac Magnetic Resonance Imaging

#### 2.5.1. Acquisition Protocol

CMR studies were performed on a 3T wide bore magnet Elition X whole-body scanner (Philips Healthcare, Best, The Netherlands) equipped with a 28-element phased-array cardiac coil. Standard segmented cine steady-state free-precession sequence (repetition time/echo time/flip angle (TR/TE/α) = 2.7 ms/1.35 ms/40°) provided high-quality anatomical references to evaluate the ventricular mass, volume, thickness, and ejection fraction. A field of view (FOV) of 320 × 320 mm, slice thickness of 8 mm with no gaps, in-plane resolution of 1.8 × 1.8 mm^2^, and 30 acquired cardiac phases were used. The 4D-flow was acquired using a 3D spoiled turbo field echo sequence (TR/TE/α = 3.6 ms/2.2 ms/7°) with an isotropic resolution of 2.5 × 2.5 × 2.5 mm^3^ and 20 acquired cardiac phases covering an imaging volume of 320 × 300 × 300 mm^3^ (FH, LR and AP direction, respectively). Images were acquired in three velocity-encoding directions and the maximum velocity encoding was adjusted according to the maximum velocity. A parallel acceleration factor of 5.7 (1.9 in the AP direction and 3 in the LR direction, respectively) was applied to reduce the total acquisition time.

#### 2.5.2. Four-Dimensional-Flow Analysis Protocol

**Figure 1:**
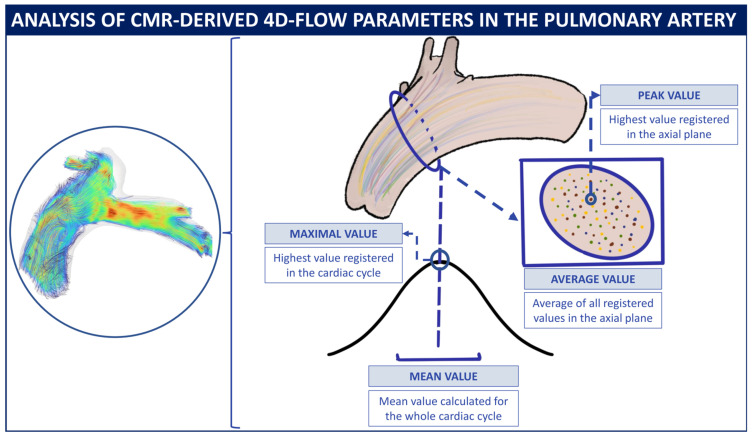
The 4D-flow sequence streamlines, segmentation, and analysis. CMR: cardiac magnetic resonance.

The 4D-flow biomarkers were calculated using an automatic tool specifically developed to analyze CMR [9]. After processing the images to correct eddy currents and velocity aliasing, it automatically segments the PA using a custom deep learning model to filter out velocity values outside the artery. It then extracts the vessel centerline and locates a perpendicular plane just above the bifurcation of the main PA anatomy (Figure 1). Using the slice of 4D flow velocity vectors projected onto that plane, it extracts the following biomarkers:

The average and peak velocity [cm/s] is defined as the mean and maximum magnitudes of the velocity vectors, respectively; flow rate [mL/s], the volume of blood passing through the plane per unit of time; average vorticity magnitude [L/s], with the vorticity defined as the curl of the velocity field and understood as a vector that describes the local flow rotation rate, has its magnitude taken and averaged across the plane; average helicity (H), with helicity being a scalar describing the relationship between flow strength and the amount of local rotation that quantifies the stability of laminar flow and its propensity toward developing turbulent structures, is averaged across the slice area; and the Reynolds number, the ratio between inertial and viscous forces, indicates flow tendency to laminar (low value) or turbulent (high value) patterns.

Average and peak velocities, flow rate, and the Reynolds number are summarized in the temporal domain with their maximum and mean across all time points. For vorticity and helicity, we used the maximum and average across the seven time points that precede the peak velocity instant, as we believed that most differences between patients occur at early diastole. For the sake of reproducibility, the formulas used to calculate these biomarkers are included in Supplementary Table S3.

### 2.6. Data Analysis

We compared the baseline characteristics, echocardiographic, and CMR-derived biomarkers between patients with IpcPH and those with CpcPH.

Continuous variables are presented as median (Q1–Q3). Categorical variables are expressed as frequencies (%). The Chi-square or Fisher’s exact test was used to compare categorical variables. The Mann–Whitney “U” test was used to compare continuous variables.

The association of 4D-flow-derived biomarkers with CpcPH was assessed using univariate logistic regression. Variables associated with CpcPH (*p* < 0.2) were selected to build a predictive model. Patient sex was also included in the model. All possible models were automatically calculated using the “allsets” command from STATA. The best model was selected based on the Akaike information criterion and the area under the curve. The accuracy of the best-selected model was compared to that of the maximal model (including all variables) by comparing the receiving operating curves.

## 3. Results

**Figure 2:**
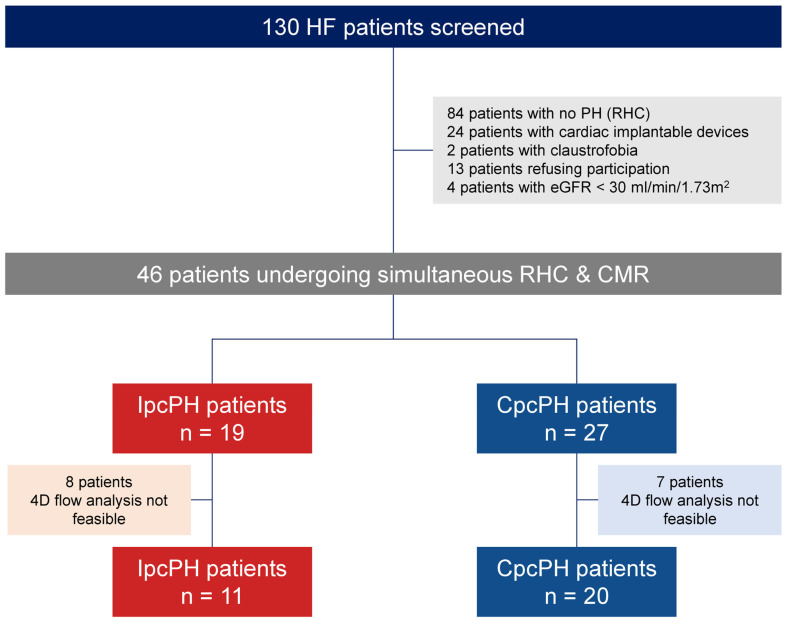
Study flowchart. CMR: cardiac magnetic resonance; CpcPH: combined postcapillary pulmonary hypertension; eGFR: estimated glomerular filtration rate; HF: heart failure; IpcPH: isolated postcapillary pulmonary hypertension PH: pulmonary hypertension.

A total of 130 patients were screened for inclusion in this study between January 2018 and December 2021. Of these, 84 were excluded after evaluating the inclusion and exclusion criteria (Figure 2).

### 3.1. Study Population and Baseline Characteristics

The study population consisted of 46 patients (55.4 ± 14 years, 63% male) who underwent RHC confirming the diagnosis of PH2 and a CMR performed within the first 24 h after RHC. In our study population, 19 (41%) patients had IpcPH and 27 (49%) had CpcPH. Table 1 summarizes the baseline characteristics of the study population and groups.

The groups were equivalent in terms of their baseline characteristics and comorbidities.

The most common HF etiology was dilated cardiomyopathy (46%), and most patients had HF with a reduced left ventricular ejection fraction (61%). Both groups were homogeneous in terms of HF etiology, left ventricular dysfunction grade, and treatment received.

### 3.2. Noninvasive Assessment and Right Heart Catheterization

Echocardiography-, CMR-, and RHC-derived parameters are detailed in Table 2. Since groups are defined based on RHC-measured pressures and pulmonary vascular resistance, the hemodynamic profile of patients with CpcPH is significantly worse than that of IpcPH patients.

No differences were found between the groups (Table 2) in echo- and CMR-based assessments.

In the CpcPH group, a non-significant higher rate of grade III diastolic dysfunction (47%-IpcPH vs. 63% CpcPH; *p* = 0.42) was found. Also, patients with CpcPH presented non-significant higher indexed RV end-diastolic (75 [51–118] mL/m^2^—IpcPH vs. 85 [65–119] mL/m^2^—CpcPH; *p* = 0.48) and end-systolic (45 [21–80] mL/m^2^—IpcPH vs. 49 [32–78] mL/m^2^—CpcPH; *p* = 0.40) volumes; and a non-significant trend toward a higher indexed RV mass (23 [19–29] g/m^2^—IpcPH. vs. 32 [29–43] g/m^2^—CpcPH; *p* = 0.11. The PA pulsatility was higher in patients with IpcPH (25.4 [15.5–31.7]—IpcPH. vs. 15.7 [9.8–26.2]—CpcPH; *p* = 0.18), but this difference was not statistically significant. RV ejection fraction and RV/PA coupling were similar in both groups.

### 3.3. Four-Dimensional-Flow-Derived Biomarkers as Predictors of CpcPH

In 15 (33%) of the 46 patients included in this study, the 4D-flow analysis could not be performed because of impossible or inappropriate acquisition.

**Figure 3:**
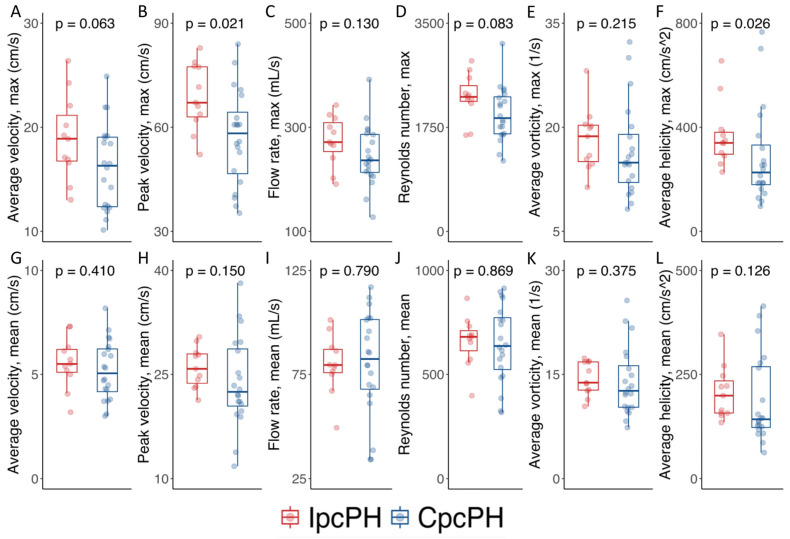
Graphical representation of 4D-derived biomarkers, maximal (**A**–**F**) and mean (**G**–**L**).

Table 3 details the 4D-flow-derived biomarkers. Figure 3 graphically represents the individual (dots) and aggregated (boxplots) values for these 4D-flow biomarkers, with maximal (Figure 3A–F) and mean (Figure 3G–L) values within the cardiac cycle.

The distribution of the mean 4D-derived biomarkers was similar between the groups (Figure 3G–L).

However, the maximal peak velocity (67.1 [62.2–77.5] cm/s—IpcPH vs. 58.2 [45.8–66.0] cm/s—CpcPH; *p* = 0.021, Figure 3B) and the maximal helicity (339.9 [290.0–391.8] cm/s^2^—IpcPH vs. 226.0 (173.5–343.7) cm/s^2^—CpcPH; *p* = 0.026, Figure 3F) were significantly lower in patients with CpcPH. The remaining maximal parameters (average velocity flow rate, Reynolds number, and average vorticity) showed non-significant differences, with a trend toward lower values in patients with CpcPH (Figure 3A,C–E).

Supplementary Table S4 shows the correlation between mean PA pressure and pulmonary vascular resistance with different 4D-flow-derived biomarkers. The mean PA pressure was not significantly correlated with any of the analyzed parameters. Pulmonary vascular resistance showed a significant negative correlation with maximal average velocity (Spearman’s rho: −0.385, *p* = 0.019), maximal peak velocity (Spearman’s rho: −0.365, *p* = 0.026), maximal flow rate (Spearman’s rho: −0.378, *p* = 0.021), maximal Reynolds number (Spearman’s rho: −0.387, *p* = 0.018), and maximal helicity (Spearman’s rho: −0.357, *p* = 0.031).

In the univariate logistic regression analysis (Table 4), only the maximal peak velocity was significantly associated with the presence of CpcPH (0.92; 95%-confidence interval: 0.86–0.99; *p* = 0.032).

**Figure 4:**
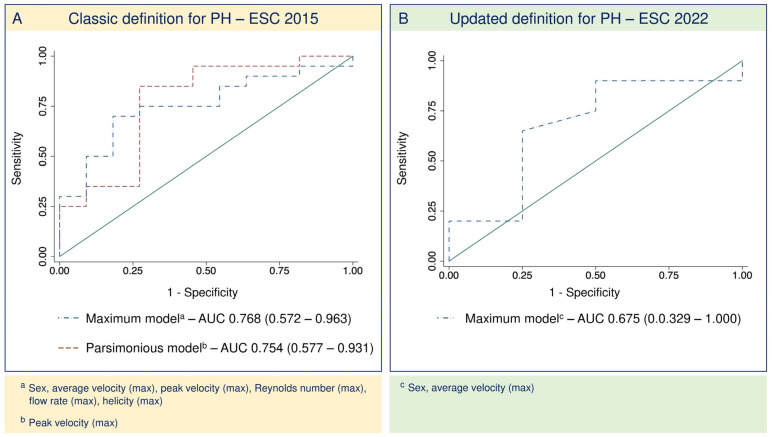
(**A**) Receiving operating curves comparing the maximal and a more parsimonious predictive model for CpcPH according to the former definition. (**B**) Receiving operation curve of the maximal predictive models for CpcPH according to the updated definition. AUC: area under the curve; PH: pulmonary hypertension; ESC: European Society of Cardiology.

A maximal predictive model was built, including the variables showing an association with a *p*-value < 0.2 (maximal average and peak velocities, maximal flow rate, maximal Reynolds number, and maximal helicity). Patient sex was also included in the model. The maximal model had an AUC of 0.768 (95% confidence interval: 0.572–0.963) with a sensitivity and specificity of 100 and 55%, respectively, and an Akaike information criterion of 46.7. Based on the smaller Akaike information criterion for the equivalent area under the curve, the best-selected model included only the peak maximal velocity. The AUC for the selected model was 0.755 (95% confidence interval: 0.578–0.931), and the sensitivity and specificity were 80% and 46%, respectively, with an Akaike information criterion of 38.3 (Figure 4A).

### 3.4. Updated Definition for PH2

When applying the updated definition of PH2 to our population, 12 patients previously classified as having IpcPH were transferred to the CpcPH group (7 [15%] IpcPH patients; 39 [85%] CpcPH patients). No significant differences were found in any of the analyzed 4D-flow biomarkers (Supplementary Table S5), and only the maximal average velocity showed an association (*p* < 0.2) in the logistic regression analysis. When studying the maximal model, we obtained an area under the curve smaller than that obtained when the classic PH2 definition was applied, with a broader confidence interval and a model’s specificity of 0% (Figure 4B).

## 4. Discussion

This study included 46 patients with PH2 who underwent simultaneous RHC and CMR to determine whether CMR-based 4D-flow analysis could help to identify HF patients who have developed CpcPH. To the best of our knowledge, this is the first study to assess the diagnostic performance of 4D-flow in PH2 patients with pulmonary vascular remodeling. The main findings of this study are (central illustration) the following: (1) IpcPH and CpcPH patients have similar characteristics, with no significant differences in clinical or noninvasive variables allowing us to distinguish both groups with currently available diagnostic tools; (2) the analysis of CMR-based 4D-flow shows that CpcPH patients present different maximal flow patterns while mean flow patterns were equivalent; (3) a maximal multiparametric model, including sex, and maximal values for peak and average velocity, Reynolds number, flow rate, and helicity presents a sensitivity and a specificity of 100% and 55%, respectively, with an AUC of 0.768; and (4) the diagnostic performance of 4D-flow lost accuracy when applying the updated definition for PH2.

### 4.1. Noninvasive Assessment of Pulmonary Circulation

RHC is the gold standard technique for the diagnosis and stratification of PH and also provides additional information about heart hemodynamics (preload, afterload, and cardiac output) [3,7]. RHC is the only diagnostic tool allowing patients to be identified with CpcPH. Although it is a safe procedure, RHC is an invasive technique that is not exempt from risks [10]. Therefore, the development of noninvasive techniques for assessing pulmonary circulation has gained interest in recent years [11].

Echocardiography, computed tomography, and CMR are the most commonly used noninvasive techniques for the evaluation of the RV and pulmonary circulation. The echocardiogram provides information about PA pressure and RV function and is a powerful screening technique for the detection of PH [3]. In addition, in the case of PH2, echocardiography is able to diagnose the cause leading to a retrograde increase in pulmonary artery pressure [3]. However, despite its capability to estimate PA pressure, the echocardiogram cannot distinguish between patients who have developed pulmonary vascular remodeling and those with isolated retrograde PH since no reliable method to estimate LV filling pressures has been properly validated [12]. In our population, the echocardiographic characteristics of patients with IpcPH and CpcPH were identical, except for the higher estimated PA systolic pressure in patients with CpcPH.

CMR offers a significant improvement in evaluating RV and pulmonary circulation compared to 2D echocardiography. It provides excellent spatial resolution of the right ventricle, evaluation of blood flow patterns in the PA, tissue characterization, and evaluation of dynamic afterload parameters [11,13]. PA rigidity and pulsatility can also be assessed by measuring the PA area throughout the cardiac cycle (11). CMR is not only useful for diagnosing PH but can also help identify RV tissue damage before the development of RV dysfunction and has been shown to predict clinical worsening and mortality in pulmonary arterial hypertension [14,15]. Also, in patients with HF and preserved ejection fraction, an increased native T1 time at the RV insertion points showed a good correlation with PA hemodynamic parameters. This is associated with poorer outcomes [16]. In our population, in patients with CpcPH, the CMR-based analysis of the RV and the pulmonary circulation was not different from that in IpcPH patients, despite having a significantly worse hemodynamic profile. Thus, despite being an excellent tool for the diagnosis, stratification, and prognostic assessment of PH, the commonly used CMR techniques cannot differentiate between IpcPH and CpcPH.

Our study results confirm that RHC is the only currently available tool for diagnosing CpcPH and further developments of new noninvasive techniques that allow simple and reliable identification of patients with PH2 and pulmonary vascular remodeling are needed.

### 4.2. Evaluation of PA Flow Patterns with 4D-Flow Sequences

Four-dimensional phase-contrast (velocity-encoded) CMR consists of a volumetric time-resolved acquisition gated to the cardiac cycle, providing a time-varying vector field of blood flow and registered anatomic images. Three-directional velocity encoding enables a more accurate analysis of the 3D-flow phenomena and the computation of secondary flow parameters [17].

Several studies evaluating 4D-flow CMR in PH have shown that patients with group 1 PH present lower PA flow velocities than healthy individuals [18,19]. Also, a significant negative correlation between 4D-derived PA flow velocity and pulmonary vascular resistance has been reported in patients with PH2 [18]. In our study population, patients with CpcPH showed a significantly lower maximal peak velocity in the main PA, and the maximal average velocity also showed a trend toward lower values in CpcPH. These findings are consistent with previously published data and suggest that 4D-flow assessed velocity might be helpful for noninvasive measurement of pulmonary vascular resistance [20].

The Reynolds number is a dimensionless quantity used in flow dynamics that helps to determine whether a flow is laminar or turbulent. Patients with PH commonly present with a dilated PA, so a more turbulent flow would be expected. However, although non-significant in our study population, patients with CpcPH had a smaller Reynolds number despite having a larger PA. This may indicate that the abnormally increased pulmonary vascular resistance, leading to a slow flow status, is not balanced by the dilatation of the PA in patients with CpcPH. In previous studies of patients with systemic sclerosis, a reduced Reynolds number was associated with poorer survival [21]. Thus, a smaller Reynolds number in patients with PH2 could be reflecting the more advanced stage of the disease in which this contradictory decrease in Reynolds number is not reflecting normal vascular physiology but a disproportionally reduced PA flow secondary to the presence of established pulmonary vasculopathy [22]. Vorticity and helicity in the main PA are strongly negatively associated with pulmonary vascular resistance [20,23]. Also, in our patients, helicity was significantly lower among those patients with CpcPH, and vorticity showed a trend toward lower values. This lower vorticity and helicity could seem counterintuitive because a more turbulent flow is expected in patients with more severe PH. However, this controversy arises from a misconception between qualitative flow analyses and quantitative spatial hemodynamic markers, such as helicity and vorticity, which are proportional to the strength and cohesiveness of the flow. Thus, the decrease in both parameters is consistent with the presence of turbulent flow, which is strongly correlated with vascular remodeling (proliferation, inflammation, and endothelial dysfunction), leading to an increased vascular stiffness [4,23].

In our study, a maximal multiparametric model including flow and velocity parameters and spatial hemodynamic parameters showed good diagnostic performance, with an area under the curve of 0.768 and a sensitivity and specificity of 100% and 55%, respectively. Thus, our study suggests that the good correlation between 4D-flow derived biomarkers and pulmonary vascular resistance makes this technique a promising tool for the noninvasive identification of HF patients with CpcPH. A more parsimonious model showed a comparable area under the curve, but worse sensitivity and specificity. However, it must be noted that all biomarkers included in the maximal model were successfully measured for all patients in whom a 4D-flow analysis was performed. Therefore, a maximal model including variables assessing both PA pressure and resistance might help to identify CpcPH without the risk of losing diagnostic capability due to missing biomarkers.

When applying the updated definition for PH and probably due to the significant reduction in the number of patients with IpcPH, 4D-flow-derived biomarkers did not show a good diagnostic performance. It must be noted that with the application of the updated definition, only six patients remained in the IpcPH group, which could have influenced the decreased diagnostic performance.

In our population, 4D-flow-derived biomarkers presented were not significantly correlated with mean PA pressure, while a significant negative correlation was found with pulmonary vascular resistance. Thus, the capacity of 4D-flow-derived biomarkers to distinguish between IpcPH and CpcPH might stem from their correlation with pulmonary vascular resistance.

### 4.3. Study Limitations

This study had several limitations that must be acknowledged. First, the recruitment rate was notably lower than expected [6]. This is explained by two facts: the difficulty in finding patients with HF with reduced ejection fraction and not having an implantable cardiac device (for safety reasons, these patients were excluded from the study because most of the devices were not tested in a 3T magnetic field), and the considerable impact the coronavirus pandemic had in the region of Madrid during the years 2020 and 2021, which drastically reduced our capability to include patients. This weakens the robustness of the obtained results, especially when group analysis was performed (IpcPH and CpcPH). This could also explain the lack of statistical significance for some of the analyzed parameters. Second, we could not obtain an appropriate 4D-flow sequence acquisition or analysis in one-third of patients. This could limit the current applicability of this technique. However, it has to be highlighted that most acquisition failures occurred during the first half of the study, reflecting the refinement of our acquisition protocol in parallel to the study run. Acquisition protocols should therefore be refined and harmonized before the introduction of this technique in daily clinical practice. Last, predictive models were proposed to illustrate the validity of the technique. However, these predictive models’ accuracy is limited by the small sample size. In fact, sensitivity and specificity are markedly imbalanced and when the updated definition for PH is applied, the accuracy of the model is smaller.

With the detailed description of all the study limitations the authors aim to highlight the exploratory character of our study. Future studies, with larger sample sizes and refined acquisition and analysis protocols are needed to validate these results.

### 4.4. Potential Translation into Clinical Practice

Invasive RHC is the only available tool for the diagnosis of CpcPH. Although relatively safe, it is not exempt from potential complications and requires dedicated and highly specialized staff and catheterization laboratory facilities. The development of imaging techniques facilitates the diagnosis of medical conditions with progressively shorter acquisition times and lower risks. In addition, the automatization of imaging analysis allows a fast and reliable measurement reducing the interobserver variability.

The translation of our study results into clinical practice is hampered by the above-mentioned limitations. However, we do believe that these results are encouraging enough to pursue future multicentric studies including a larger population aiming to validate the conclusions obtained from this single-center pilot study.

## 5. Conclusions

The analysis of CMR-based 4D-flow of the pulmonary artery could be a useful technique for the identification of patients with CpcPH. The 4D-flow-derived maximal peak velocity and maximal helicity were significantly lower in patients with CpcPH. A multiparametric model including several maximal 4D-flow-derived biomarkers showed good diagnostic performance for the identification of PH2 patients with CpcPH. Future studies with larger populations and based on the updated definition of PH2 and CpcPH are warranted to confirm the role of CMR-based 4D-flow analysis in identifying pulmonary vascular remodeling.

## Figures and Tables

**Table 1:** Baseline characteristics.

	Total	IpcPH	CpcPH	*p*-Value
	N = 46	N = 19	N = 27	
Baseline characteristics				
Age, years	55.4 (14.0)	55.8 (14.9)	55.2 (13.5)	0.89
Male sex, n (%)	29 (63%)	13 (68%)	16 (59%)	0.53
BMI, kg/m^2^	27.2 (5.2)	28.4 (5.0)	26.3 (5.3)	0.19
Hypertension, n (%)	23 (50%)	7 (37%)	16 (59%)	0.13
Dyslipidemia, n (%)	17 (37%)	5 (26%)	12 (44%)	0.21
Ischemic heart disease, n (%)	10 (22%)	2 (11%)	8 (30%)	0.12
Chronic kidney disease, n (%)	16 (35%)	4 (21%)	12 (44%)	0.10
eGFR, mL/min/1.73 m^2^	68.5 (18.4)	74.5 (18.5)	64.4 (17.5)	0.066
Respiratory disease	9 (20%)	4 (21%)	5 (19%)	0.19
COPD, n (%)	3 (7%)	3 (16%)	0 (0%)	
OSA, n (%)	4 (9%)	1 (5%)	3 (11%)	
Restrictive, n (%)	1 (2%)	0 (0%)	1 (4%)	
Others, n (%)	1 (2%)	0 (0%)	1 (4%)	
FVC, %	75.3 (14.8)	73.1 (13.1)	76.6 (16.0)	0.51
FEV1, %	70.6 (18.0)	65.6 (19.6)	73.5 (16.8)	0.21
DLCO-VA, %	79.6 (12.8)	80.6 (14.3)	79.1 (12.3)	0.76
Atrial fibrillation, n (%)	19 (41%)	9 (47%)	10 (37%)	0.48
Heart failure characteristics				
Etiology				0.25
Ischemic, n (%)	10 (22%)	2 (11%)	8 (30%)	
Hypertrophic, n (%)	1 (2%)	0 (0%)	1 (4%)	
Dilated, n (%)	21 (46%)	12 (63%)	9 (33%)	
Restrictive, n (%)	4 (9%)	2 (11%)	2 (7%)	
Other, n (%)	10 (22%)	3 (16%)	7 (26%)	
HF classification LVEF, n (%)				0.53
HFrEF, n (%)	28 (61%)	13 (68%)	15 (56%)	
HFmrEF, n (%)	5 (11%)	1 (5%)	4 (15%)	
HFpEF, n (%)	13 (28%)	5 (26%)	8 (30%)	
NYHA functional class				
II, n (%)	24 (52%)	8 (42%)	16 (59%)	0.42
III, n (%)	19 (41%)	9 (47%)	10 (37%)	
IV, n (%)	3 (7%)	2 (11%)	1 (4%)	
NT-proBNP, pg/mL	2043.0 (1136.0–3242.0)	1751.0 (1338.0–4000.0)	2074.0 (939.0–3114.0)	0.83
HF hospitalization, n (%)	20 (43%)	7 (37%)	13 (48%)	0.45
Heart failure treatment				
ACEI/ARB, n (%)	22 (48%)	12 (63%)	10 (37%)	0.081
ARNI, n (%)	10 (22%)	4 (22%)	6 (22%)	1.00
MRA, n (%)	33 (72%)	16 (84%)	17 (63%)	0.12
Beta-blockers, n (%)	38 (83%)	18 (95%)	20 (74%)	0.069
Ivabradine, n (%)	8 (17%)	4 (21%)	4 (15%)	0.58
Loop diuretic, n (%)	40 (87%)	16 (84%)	24 (89%)	0.64
Furosemide, n (%)	38 (83%)	15 (79%)	23 (85%)	0.58
Furosemide dose, mg/day	40 (40–80)	40 (40–80)	50 (40–80)	0.69
Torasemide, n (%)	2 (4%)	1 (5%)	1 (4%)	0.80
Torasemide dose, mg/day	10	10	10	NA

ACEI/ARB: angiotensin converter enzyme inhibitors/angiotensin receptor blockers; ARNI: angiotensin receptor neprilisin inhibitor; BMI: body mass index; COPD: chronic obstructive pulmonary disease; DLCO-VA: diffusing capacity of the lung for carbon monoxide, adjusted by alveolar volume; FEV1: forced expiratory volume in 1 s; FVC: forced vital capacity; eGFR: estimated glomerular filtration rate; HF: heart failure; HFmrEF: heart failure with mildly reduced ejection fraction; HFpEF: heart failure with preserved ejection fraction; HFrEF: heart failure with reduced ejection fraction; MRA: mineralocorticoid receptor antagonist; NYHA: New York Heart Association; OSA: obstructive sleep apnea.

**Table 2:** Echo- and CMR- and RHC-derived parameters.

	Total	IpcPH	CpcPH	*p*-Value
	N = 46	N = 19	N = 27	
Echocardiography parameters				
LVEF, %	38 (25–56)	33 (24–56)	39 (28–56)	0.50
Diastolic dysfunction	32 (70%)	12 (63%)	20 (74%)	0.42
Grade I, n (%)	2 (4%)	1 (5%)	1 (4%)	
Grade II, n (%)	4 (9%)	2 (11%)	2 (7%)	
Grade III, n (%)	26 (57%)	9 (47%)	17 (63%)	
E/A ratio	3.0 (2.2–3.5)	3.2 (2.0–3.6)	2.9 (2.3–3.4)	0.96
E/e’ ratio	12.1 (7.8–15.3)	10.0 (6.8–14.0)	13.3 (9.5–15.3)	0.27
Indexed LA volume, cm^3^/m^2^	31 (25–39)	28 (23–38)	33 (26–44)	0.30
Right ventricular dysfunction				0.36
Mild, n (%)	11 (24%)	2 (11%)	9 (33%)	
Moderate, n (%)	10 (22%)	5 (26%)	5 (19%)	
Severe, n (%)	4 (9%)	2 (11%)	2 (7%)	
TAPSE, mm	17.0 (15.0–20.0)	16.0 (15.0–19.0)	18.0 (15.0–20.0)	0.18
Mitral regurgitation (>mild), n (%)	14 (30%)	5 (26%)	9 (33%)	0.61
Tricuspid regurgitation (>mild), n (%)	10 (22%)	4 (21%)	6 (22%)	0.92
Systolic PA pressure, mmHg	55 (50–63)	50.5 (44.5–55.5)	61 (54–65)	0.01
CMR parameters				
*Left chambers*				
LVEF, %	34 (20–50)	27 (19–50)	36 (21–54)	0.40
LA area, cm^2^	36 (30–43)	39 (32–43)	35 (28–42)	0.26
Indexed end-diastolic LV volume, mL/m^2^	109 (83–159)	130 (75–169)	100 (83–147)	0.35
Indexed end-systolic LV volume, mL/m^2^	70 (43–115)	93 (44–137)	64 (38–112)	0.35
Indexed LV mass, g/m^2^	73 (56–82)	68 (62–87)	73 (55–80)	0.47
Indexed LV cardiac output, mL/min/m^2^	1.9 (1.6–2.4)	1.7 (1.6–2.0)	2.2 (1.6–2.6)	0.07
*Right chambers*				
RA area, cm^2^	27 (21–30)	28 (20–32)	24 (21–30)	0.58
RVEF, %	40 (30–54)	41 (27–61)	40 (30–52)	0.78
Indexed RV end-diastolic volume, mL/m^2^	80 (64–118)	75 (51–118)	85 (65–119)	0.48
Indexed RV end-systolic volume, mL/m^2^	46 (32–78)	45 (21–80)	49 (32–78)	0.40
Indexed RV mass, g/m^2^	29 (21–32)	23 (19–29)	32 (29–43)	0.11
Indexed RV cardiac output, mL/min/m^2^	2.0 (1.6–2.4)	2.0 (1.6–2.2)	2.0 (1.6–2.9)	0.42
PA diameter, mm	28 (27–31)	28 (24.5–28)	31 (28–33)	0.01
PA pulsatility	19.9 (10.3–30.9)	25.4 (15.5–31.7)	15.7 (9.8–26.2)	0.18
RV/PA coupling	1.6 (1.1–2.4)	1.6 (0.8–2.6)	1.6 (1.1–2.3)	0.94
RHC parameters				
Right atrial pressure, mmHg	11 (8–13)	12 (9–15)	10 (7–11)	0.05
Systolic PA pressure, mmHg	58 (50–69)	48 (39–56)	65 (56–75)	<0.001
Diastolic PA pressure, mmHg	24 (21–29)	23 (21–27)	25 (20–33)	0.58
Mean PA pressure, mmHg	38 (32–43)	32 (30–38)	41 (37–46)	<0.001
PAWP, mmHg	22 (19–27)	23 (21–30)	22 (18–26)	0.07
Cardiac index, L/min/m^2^	2.2 (1.9–2.6)	2.2 (1.9–2.5)	2.3 (1.9–2.7)	0.99
Pulmonary vascular resistance, WU	4 (2–5)	2 (1–3)	5 (4–6)	<0.001
Transpulmonary gradient *, mmHg	15 (9–20)	9 (7–11)	20 (17–24)	<0.001

LA: left atrium; LV: left ventricle; LVEF: left ventricle ejection fraction; PA: pulmonary artery; PAWP: pulmonary artery wedge pressure; RA: right atrium; RV: right ventricle; RVEF: right ventricle ejection fraction; TAPSE: tricuspid annulus plane systolic excursion. * Transpulmonary gradient = Mean PA pressure—PAWP.

**Table 3:** The 4D-flow parameters.

	Total	IpcPH	CpcPH	*p*-Value
	N = 31	N = 11	N = 20	
Average velocity (max), cm/s	16.9 (13.0–19.2)	18.9 (16.6–22.1)	16.3 (12.4–19.1)	0.063
Average velocity (mean), cm/s	5.4 (4.3–6.3)	5.5 (5.0–6.3)	5.1 (4.1–6.2)	0.410
Peak velocity (max), cm/s	60.9 (52.8–71.6)	67.1 (62.2–77.5)	58.2 (45.8–66.0)	0.021
Peak velocity (mean), cm/s	24.2 (21.1–28.4)	25.8 (23.2–28.1)	22.5 (20.2–29.0)	0.150
Flow rate (max), mL/s	254.8 (214.8–294.7)	271.4 (241.2–317.5)	236.4 (211.6–286.7)	0.130
Flow rate (mean), mL/s	79.5 (68.9–96.9)	79.5 (75.1–87.9)	82.4 (67.0–101.6)	0.790
Reynolds number (max)	2151.0 (1643.6–2323.2)	2262.2 (2151.0–2580.7)	1905.0 (1634.8–2279.4)	0.083
Reynolds number (mean)	672.0 (533.6–759.9)	680.4 (572.7–731.0)	636.3 (512.6–781.4)	0.869
Average vorticity (max), L/s	15.7 (13.0–20.2)	18.7 (14.7–20.4)	14.9 (11.8–19.4)	0.215
Average vorticity (mean), L/s	13.3 (10.4–16.8)	13.8 (12.7–16.8)	12.6 (10.2–16.7)	0.375
Average helicity (max), cm/s^2^	270.7 (186.4–369.8)	339.9 (290.0–391.8)	226.0 (173.5–343.7)	0.026
Average helicity (mean), cm/s^2^	157.2 (128.0–265.4)	199.2 (157.2–246.5)	142.3 (121.5–271.5)	0.126

**Table 4:** Logistic regression analysis evaluating the association of 4D-flow derived biomarkers with CpcPH.

Variable	OR (95%—CI)	*p*-Value
Maximal average velocity, cm/s	0.84 (0.69–1.03)	0.090
Mean average velocity, cm/s	0.82 (0.46–1.43)	0.469
Maximal peak velocity, cm/s	0.92 (0.86–0.99)	0.032
Mean peak velocity, cm/s	0.93 (0.81–1.07)	0.316
Maximal flow rate, cm/s	0.99 (0.98–1.00)	0.176
Mean flow rate, cm/s	1.00 (0.97–1.04)	0.934
Maximal Reynolds number	0.99 (0.99–1.00)	0.096
Mean Reynolds number	1.00 (1.00–1.00)	0.792
Maximal average vorticity, L/s	0.95 (0.84–1.08)	0.466
Mean average vorticity, L/s	0.98 (0.82–1.16)	0.794
Maximal average helicity, cm/s^2^	1.00 (0.99–1.00)	0.190
Mean average helicity, cm/s^2^	1.00 (0.99–1.01)	0.593

OR: Odds ratio; CI: confidence interval.

## Data Availability

The data that support the findings of this study are available on request from the corresponding author, [J.F.D].

## Supplementary Materials

The following supporting information can be downloaded at: https://www.mdpi.com/article/10.3390/jcm14030929/s1, Figure S1: Study design; Table S1. Former and current definitions of group 2 pulmonary hypertension; Table S2: Inclusion and exclusion criteria; Table S3: Formulas for biomarkers calculation; Table S4: Correlation between 4D-based biomarkers and mean pulmonary artery pressure and pulmonary vascular resistance; Table S5: 4D-based biomarkers distribution according to the updated definition for pulmonary hypertension.

## Abbreviations

AUC	Area under the curve
CMR	Cardiac magnetic resonance
CpcPH	Combined postcapillary pulmonary hypertension
HF	Heart Failure
IpcPH	Isolated postcapillary pulmonary hypertension
PH	Pulmonary hypertension
PH2	Group 2 pulmonary hypertension
RHC	Right Heart Catheterization
RV	right ventricle

## References

[1] Roger V.L. (2021). Epidemiology of Heart Failure: A Contemporary Perspective. Circ. Res..

[2] Vachiéry J.L., Tedford R.J., Rosenkranz S., Palazzini M., Lang I., Guazzi M., Coghlan G., Chazova I., De Marco T. (2019). Pulmonary hypertension due to left heart disease. Eur. Respir. J..

[3] Humbert M., Kovacs G., Hoeper M.M., Badagliacca R., Berger R.M.F., Brida M., Carlsen J., Coats A.J.S., Escribano-Subias P., Ferrari P. (2022). 2022 ESC/ERS Guidelines for the diagnosis and treatment of pulmonary hypertension. Eur. Heart J..

[4] Humbert M., Guignabert C., Bonnet S., Dorfmüller P., Klinger J.R., Nicolls M.R., Olschewski A.J., Pullamsetti S.S., Schermuly R.T., Stenmark K.R. (2019). Pathology and pathobiology of pulmonary hypertension: State of the art and research perspectives. Eur. Respir. J..

[5] Gerges M., Gerges C., Pistritto A.M., Lang M.B., Trip P., Jakowitsch J., Binder T., Lang I.M. (2015). Pulmonary Hypertension in Heart Failure. Epidemiology, Right Ventricular Function, and Survival. Am. J. Respir. Crit. Care Med..

[6] Ponz I., Nuche J., Sanchez Sanchez V., Sanchez-Gonzalez J., Blazquez-Bermejo Z., Caravaca Perez P., Garcia-Cosio Carmena M.D., de Juan Baguda J.S., Rodríguez Chaverri A., Sarnago Cebada F. (2021). Non-Invasive Assessment of Pulmonary Vasculopathy. Hearts.

[7] Galiè N., Humbert M., Vachiery J.L., Gibbs S., Lang I., Torbicki A., Simonneau G., Peacock A., Vonk Noordegraaf A., Beghetti M. (2016). 2015 ESC/ERS Guidelines for the diagnosis and treatment of pulmonary hypertension: The Joint Task Force for the Diagnosis and Treatment of Pulmonary Hypertension of the European Society of Cardiology (ESC) and the European Respiratory Society (ERS): Endorsed by: Association for European Paediatric and Congenital Cardiology (AEPC), International Society for Heart and Lung Transplantation (ISHLT). Eur. Heart J..

[8] Ponikowski P., Voors A.A., Anker S.D., Bueno H., Cleland J.G.F., Coats A.J.S., Falk V., González-Juanatey J.R., Harjola V.P., Jankowska E.A. (2016). 2016 ESC Guidelines for the diagnosis and treatment of acute and chronic heart failure: The Task Force for the diagnosis and treatment of acute and chronic heart failure of the European Society of Cardiology (ESC)Developed with the special contribution of the Heart Failure Association (HFA) of the ESC. Eur. Heart J..

[9] Bóbeda J., Erostarbe H., Stephens M., Gaitán Á., Kumar R., Nuche J., Marco I., Delgado J., Ruíz-Cabello J., López-Linares K. Automatic Tool for Pulmonary Artery Hemodynamic Assessment from 4D flow MRI. Proceedings of the 2023 IEEE 20th International Symposium on Biomedical Imaging (ISBI).

[10] Hoeper M.M., Lee S.H., Voswinckel R., Palazzini M., Jais X., Marinelli A., Barst R.J., Ghofrani H.A., Jing Z.C., Opitz C. (2006). Complications of right heart catheterization procedures in patients with pulmonary hypertension in experienced centers. J. Am. Coll. Cardiol..

[11] Hur D.J., Sugeng L. (2019). Non-invasive Multimodality Cardiovascular Imaging of the Right Heart and Pulmonary Circulation in Pulmonary Hypertension. Front. Cardiovasc. Med..

[12] Martelli G., Congedi S., Lorenzoni G., Nardelli M., Lucchetta V., Gregori D., Tiberio I. (2023). Echocardiographic assessment of pulmonary capillary wedge pressure by E/e’ ratio: A systematic review and meta-analysis. J. Crit. Care.

[13] Sanz J., García-Alvarez A., Fernández-Friera L., Nair A., Mirelis J.G., Sawit S.T., Pinney S., Fuster V. (2012). Right ventriculo-arterial coupling in pulmonary hypertension: A magnetic resonance study. Heart.

[14] Alabed S., Shahin Y., Garg P., Alandejani F., Johns C.S., Lewis R.A., Condliffe R., Wild J.M., Kiely D.G., Swift A.J. (2021). Cardiac-MRI Predicts Clinical Worsening and Mortality in Pulmonary Arterial Hypertension: A Systematic Review and Meta-Analysis. JACC Cardiovasc. Imaging.

[15] García-Álvarez A., García-Lunar I., Pereda D., Fernández-Jimenez R., Sánchez-González J., Mirelis J.G., Nuño-Ayala M., Sánchez-Quintana D., Fernández-Friera L., García-Ruiz J.M. (2015). Association of myocardial T1-mapping CMR with hemodynamics and RV performance in pulmonary hypertension. JACC Cardiovasc. Imaging.

[16] Nitsche C., Kammerlander A.A., Binder C., Duca F., Aschauer S., Koschutnik M., Snidat A., Beitzke D., Loewe C., Bonderman D. (2019). Native T1 time of right ventricular insertion points by cardiac magnetic resonance: Relation with invasive haemodynamics and outcome in heart failure with preserved ejection fraction. Eur. Heart J. Cardiovasc. Imaging.

[17] Barker A.J., Roldán-Alzate A., Entezari P., Shah S.J., Chesler N.C., Wieben O., Markl M., François C.J. (2015). Four-dimensional flow assessment of pulmonary artery flow and wall shear stress in adult pulmonary arterial hypertension: Results from two institutions. Magn. Reson. Med..

[18] Cerne J.W., Pathrose A., Gordon D.Z., Sarnari R., Veer M., Blaisdell J., Allen B.D., Avery R., Markl M., Ragin A. (2022). Evaluation of Pulmonary Hypertension Using 4D Flow MRI. J. Magn. Reson. Imaging.

[19] Odagiri K., Inui N., Hakamata A., Inoue Y., Suda T., Takehara Y., Sakahara H., Sugiyama M., Alley M.T., Wakayama T. (2016). Non-invasive evaluation of pulmonary arterial blood flow and wall shear stress in pulmonary arterial hypertension with 3D phase contrast magnetic resonance imaging. SpringerPlus.

[20] Kheyfets V.O., Schafer M., Podgorski C.A., Schroeder J.D., Browning J., Hertzberg J., Buckner J.K., Hunter K.S., Shandas R., Fenster B.E. (2016). 4D magnetic resonance flow imaging for estimating pulmonary vascular resistance in pulmonary hypertension. J. Magn. Reson. Imaging.

[21] Ikoma T., Suwa K., Sano M., Ushio T., Saotome M., Ogawa N., Satoh H., Maekawa Y. (2021). Early changes of pulmonary arterial hemodynamics in patients with systemic sclerosis: Flow pattern, WSS, and OSI analysis with 4D flow MRI. Eur. Radiol..

[22] Fayyaz A.U., Edwards W.D., Maleszewski J.J., Konik E.A., DuBrock H.M., Borlaug B.A., Frantz R.P., Jenkins S.M., Redfield M.M. (2018). Global Pulmonary Vascular Remodeling in Pulmonary Hypertension Associated With Heart Failure and Preserved or Reduced Ejection Fraction. Circulation.

[23] Schäfer M., Barker A.J., Kheyfets V., Stenmark K.R., Crapo J., Yeager M.E., Truong U., Buckner J.K., Fenster B.E., Hunter K.S. (2017). Helicity and Vorticity of Pulmonary Arterial Flow in Patients with Pulmonary Hypertension: Quantitative Analysis of Flow Formations. J. Am. Heart Assoc..

