# Supplementary material for: Four-Dimensional Magnetic Resonance Pulmonary Flow Imaging for Assessing Pulmonary Vasculopathy in Patients with Postcapillary Pulmonary Hypertension
Source: J Clin Med. 2025 Jan 31;14(3):929. doi: 10.3390/jcm14030929 (PMC11818231; doi:10.3390/jcm14030929)
Supplement: Supplementary file 1 [file jcm-14-00929-s001.zip › jcm-3409158-supplementary.pdf]

4D – magnetic resonance pulmonary flow imaging for assessing pulmonary vasculopathy in patients with postcapillary pulmonary hypertension.

SUPPLEMENTARY MATERIAL

Detailed CMR acquisition protocol:

CMR studies were performed on a 3T wide bore magnet Elition X whole-body scanner (Philips Healthcare, Best, The Netherlands) equipped with a 28-element phased-array cardiac coil. Standard segmented cine steady-state free-precession sequence (repetition time/echo time/flip angle (TR/TE/α) = 2.7 ms/1.35 ms/40◦) provided high-quality anatomical references to evaluate the ventricular mass, volume, thickness, and ejection fraction. Field of view (FOV) of 320 × 320 mm, slice thickness of 8 mm with no gaps, and in-plane resolution of 1.8 × 1.8 mm2 and 30 acquired cardiac phases were used. 4D-flow was acquired using a 3D spoiled turbo field echo sequence (TR/TE/α =3.6ms/2.2ms/7º) with isotropic resolution of 2.5x2.5x2.5 mm^3 and 20 acquired cardiac phases covering an imaging volume of 320x300x300 mm^3 (FH, LR and AP direction respectively). Images were acquired in three velocity-encoding directions and the maximum velocity encoding was adjusted according to the maximum velocity. A parallel acceleration factor of 5.7 (1.9 in the AP direction and 3 in the LR direction, respectively) was applied to reduce the total acquisition time.

Supplementary table S1. Former and current definitions of group 2 pulmonary hypertension.

	**Former definition (2015 – ESC guidelines)**	**Current definition (2022 – ESC guidelines)**
**Post-capillary PH**	MPAP ≥ 25 mmHg<br>PCWP > 15 mmHg	MPAP > 20 mmHg<br>PCWP > 15 mmHg
**IpcPH**	DPG < 7 mmHg and/or<br>PVR ≤ 3 Wood units	PVR ≤ 2 Wood units
**CpcPH**	DPG ≥ 7 mmHg and/or<br>PVR > 3 mmHg	PVR > 2 Wood units

CpcPH: combined post-capillary pulmonary hypertension; ESC: European Society of Cardiology; IpcPH: isolated postcapillary pulmonary hypertension; PAP: Mean pulmonary artery pressure; PH: pulmonary hypertension; PVR: pulmonary vascular resistance

Supplementary table S2. Inclusion and exclusion criteria.

**Inclusion criteria**
- Patients with symptomatic heart failure:<br>o NYHA functional class II-IV.<br>o Symptoms due to congestion or low output.<br>o Echocardiogramm 6 months prior to enrollment with at least 1 of the following:<br>▪ LVEF < 40% (HFrEF).<br>▪ LVEF 40-49% (HFmrEF) or ≥ 50% (HFpEF) and 1 of the following:<br>• Left ventricular hypertrophy (mass index ≥ 115 g/m2) and/or left atrial enlargement (volume index > 34 ml/m^2).<br>• Diastolic dysfunction (E/e' ≥ 13, e' septal and lateral wall < 9 cm/seg).<br>o Elevated natriuretic peptides (BNP > 35 pg/ml and/or NT-proBNP >125 pg/mL).<br>o Optimal medical and device therapy.
- PH confirmed by RHC.
- Inpatients as well as outpatients.
- Age ≥ 18 years
**Exclusion criteria**
- Pulmonary hypertension WHO group 1 or 3-5
- Congenital heart disease
- Claustrophobia
- Permanent pacemaker or an implantable cardioverter defibrillator
- Other metallic devices or prosthetic material non compatible with MRI
- Unstable clinical or hemodynamic situation not allowing to perform MRI and PFT.
- Glomerular filtration rate < 30 ml/min/1.73 m^2

HRmrEF: heart failure with mid-range ejection fraction; HFpEF: heart failure with preserved ejection fraction; HRrEF: heart failure with reduced ejection fraction; MRI: magnetic resonance imaging; PFT: pulmonary function test; PH: pulmonary hypertension; RHC: right heart catheterization; WHO: world health organization.

Supplementary figure S1. Study design.

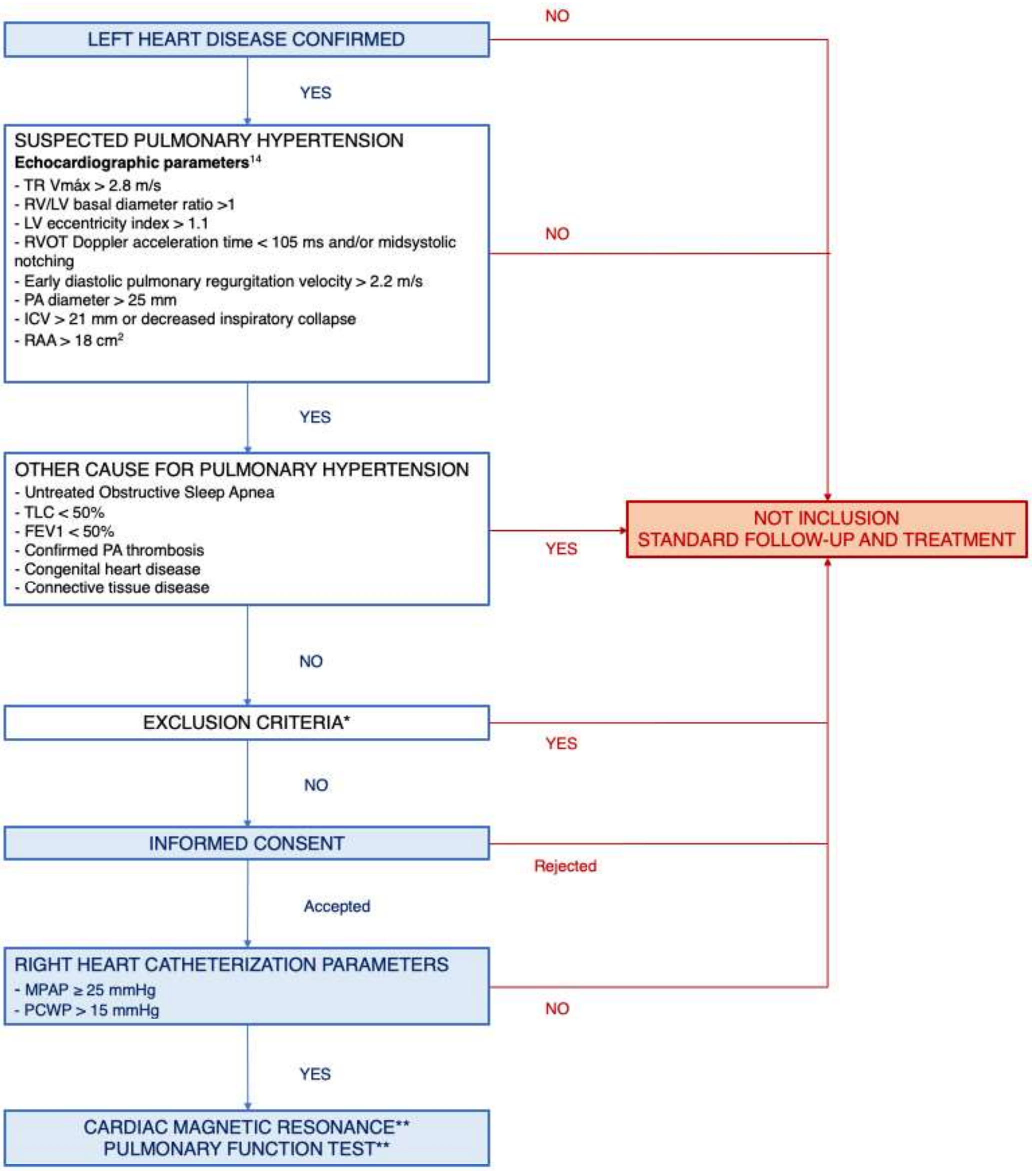

* See supplemental table 2.
** Magnetic resonance and pulmonary function test performed within 24 after catheterization
AT=anaerobic threshold; CPET=cardiopulmonary stress test; $EqCO_2$= CO_2 equivalents ICV=inferior cava vein; LV=Left ventricle; MPAP=mean pulmonary artery pressure; PA=pulmonary artery;PCWP=pulmonary capillary wedge pressure; $PetCO_2$=End-espiratory CO_2 pressure; RAA=right atrial area; RV=Right ventricle; RVOT=right ventricle outflow tract; TLC=total lung capacity; FEV1=forced expiratory volume in first second; TR=Tricuspid regurgitation

Supplementary table S3. Formulas for biomarkers calculation, $\vec{v}$ is the flow velocity vector, N the number of measurements in a slice, ∇ the spatial derivative operator Nabla, A the cross-sectional area of the artery slice, ρ the density of the fluid, V the average velocity magnitude, D the diameter of the vessel and μ the viscosity of the fluid.

**Biomarker**	**Formula**
Flow rate	$FR = \int_A \vec{v}\ dA$
Vorticity	$\vec{w} = \nabla \times \vec{v}$
Average vorticity magnitude	$W = \frac{1}{N}\sum_{i=1}^{N} \lvert\vec{w}\rvert_i$
Average helicity	$H = \frac{1}{N}\sum_{i=1}^{N} (\vec{v} \cdot \vec{\omega})_i$
Reynolds number	$\mathrm{ReN} = \frac{\rho \cdot \mathrm{V} \cdot \mathrm{D}}{\mu}$

Supplementary table S4. Correlation between 4D-based biomarkers and mean pulmonary artery pressure and pulmonary vascular resistance.

	Mean PA pressure		Pulmonary vascular resistance	
	Spearman's rho	p-value	Spearman's rho	p-value
**Average velocity (max), cm/s**	-0.056	0.743	-0.385	0.019
**Average velocity (mean), cm/s**	0.109	0.519	-0.188	0.266
**Peak velocity (max), cm/s**	0.009	0.954	-0.365	0.026
**Peak velocity (mean), cm/s**	0.041	0.812	-0.210	0.213
**Flow rate (max), ml/s**	-0.081	0.634	-0.378	0.021
**Flow rate (mean), ml/s**	0.104	0.540	0.000	0.999
**Reynold's number (max)**	-0.054	0.757	-0.387	0.018
**Reynold's number (mean)**	0.141	0.405	-0.065	0.703
**Average vorticity (max), 1/s**	0.032	0.852	-0.159	0.346
**Average vorticity (mean), 1/s**	0.129	0.445	-0.083	0.623
**Average helicity (max), cm/s2**	0.006	0.971	-0.357	0.031
**Average helicity (mean), cm/s2**	0.095	0.576	-0.196	0.244

Supplementary table S5. 4D-based biomarkers distribution according to the updated definition for pulmonary hypertension. Logistic regression analysis for association with the presence of combined postcapillary pulmonary hypertension.

	Total	IpcPH	CpcPH	p-value	OR (95% CI)	p-value
	N=31	N=4	N=27			
**Average velocity (max), cm/s**	16.9 +(13.0-19.2)	20.7 (16.7-23.2)	16.6 (12.6-19.2)	0.13	0.83 (0.64-1.09)	0.178
**Average velocity (mean), cm/s**	5.4 (4.3-6.3)	5.8 (5.2-6.7)	5.3 (4.1-6.3)	0.28	0.64 (0.28 - 1.49)	0.305
**Peak velocity (max), cm/s**	60.9 (52.8-71.6)	70.7 (57.9-78.1)	60.8 (52.8-70.8)	0.29	0.95 (0.87 - 1.04)	0.291
**Peak velocity (mean), cm/s**	24.2 (21.1-28.4)	25.3 (24.0-26.7)	23.4 (21.1-29.5)	0.64	0.97 (0.80 - 1.18)	0.794
**Flow rate (max), ml/s**	254.8 (214.8-294.7)	270.8 (215.9-321.5)	254.8 (214.8-290.5)	0.60	0.99 (0.98 - 1.01)	0.603
**Flow rate (mean), ml/s**	79.5 (68.9-96.9)	77.3 (71.1-88.2)	79.6 (68.9-101.2)	0.68	1.00 (0.95-1.05)	0.953
**Reynold's number (max)**	2151.0 (1643.6-2323.2)	2437.7 (1955.8-2647.5)	2140.8 (1643.6-2311.9)	0.26	1.00 (1.00 - 1.00)	0.284
**Reynold's number (mean)**	672.0 (555.3-759.9)	705.7 (626.6-743.8)	654.2 (533.6-764.0)	0.68	1.00 (1.00 - 1.01)	0.640
**Average vorticity (max), 1/s**	15.7 (13.0-20.2)	17.6 (15.0-20.1)	15.7 (12.3-20.2)	0.56	0.99 (0.83 - 1.17)	0.875
**Average vorticity (mean), 1/s**	13.3 (10.4-16.8)	13.2 (12.1-15.2)	13.3 (10.4-16.9)	0.98	1.03 (0.79 - 1.34)	0.823
**Average helicity (max), cm/s2**	270.7 (186.4-369.8)	344.7 (321.0-370.7)	255.1 (184.6-369.8)	0.19	1.00 (0.99 - 1.00)	0.697
**Average helicity (mean), cm/s2**	157.2 (128.0-265.4)	181.8 (160.8-209.8)	151.3 (127.2-270.7)	0.52	1.00 (0.99 - 1.01)	0.824
